# Astrocyte-selective AAV gene therapy through the endogenous GFAP promoter results in robust transduction in the rat spinal cord following injury

**DOI:** 10.1038/s41434-019-0075-6

**Published:** 2019-04-08

**Authors:** Jarred M. Griffin, Barbara Fackelmeier, Dahna M. Fong, Alexander Mouravlev, Deborah Young, Simon J. O’Carroll

**Affiliations:** 1 Centre for Brain Research, University of Auckland, Auckland, New Zealand; 2 Department of Anatomy and Medical Imaging, School of Medical Sciences, Faculty of Medical and Health Sciences, University of Auckland, Auckland, New Zealand; 3 Department of Pharmacology and Clinical Pharmacology, School of Medical Sciences, Faculty of Medical and Health Sciences, University of Auckland, Auckland, New Zealand

**Keywords:** Neuroscience, Genetic transduction

## Abstract

Adeno-associated viral (AAV) vectors are a promising system for transgene delivery into the central nervous system (CNS) based on their safety profile and long-term gene expression. Gene delivery to the CNS has largely been neuron centric but advances in AAV technology are facilitating the development of approaches to enable transduction of glial cells. Considering the role of astrocytes in the on-going secondary damage in spinal cord injury (SCI), an AAV vector that targets astrocytes could show benefit as a potential treatment. Transduction efficiency, transgene expression and cellular tropism were compared for the AAV serotypes AAV5, AAV9 and AAVRec2 whereby destabilised yellow fluorescent protein (dYFP) was controlled by the GFAP or the truncated GfaABC_1_D promoter. The vectors were tested in primary spinal cord astrocyte cell culture, spinal cord slice culture and an in vivo model of SCI contusion. AAV5 resulted in greater transduction efficiency, transgene expression and astrocyte tropism compared with AAV9 and AAVRec2. In a rodent model of SCI, robust transgene expression by AAV5-GFAP/GfaABC_1_D-dYFP was observed through 12 mm of spinal cord tissue and expression was largely restricted to astrocytes. Thus, AAV5-GFAP/GfaABC_1_D carries the potential as a potential gene therapy vector, particularly for transducing astrocytes in the damaged spinal cord.

## Introduction

Once considered passive framework cells, glial cells are now known to be key responders to central nervous system (CNS) injury and equal the number of neurons in the adult human brain [1, 2]. Furthermore, astrocytes are the most abundant glial cell type in the CNS and they play a crucial role in the pathogenesis of spinal cord injury (SCI) [3]. Gene delivery towards astrocytes may be beneficial in addressing their role in SCI. Adeno-associated viral (AAV) vector gene therapy, in particular, has emerged as the vector of choice for safe, robust and long-term transgene expression in the CNS. However, despite the importance of astrocytes in SCI, AAV vector delivery systems have been largely neuron centric and as such, the application of AAV vectors to deliver transgenes to astrocytes in spinal cord tissue is a relatively unexplored avenue.

In the brain, combining vector serotypes that have a natural propensity toward astrocyte transduction with astrocyte-specific promoters can enhance transgene expression in astrocytes while reducing the expression in neuronal populations [4]. For example, with the AAV serotypes rh43 and AAV8, enhanced Green Flourescent Protein (EGFP) expression under the control of a constitutively active, CAG promoter showed largely astrocytic transduction in brains of non-human primates [5]. These authors then went on to show that using a glial fibrillary acidic protein (GFAP) promoter, astrocyte targeting of rh43 and AAV8 could be increased to over 88% and interestingly, the total number of transduced cells nearly doubled—indicating that coupling particular capsid tropism to particular promoters is not only capable but advantageous [5].

The serotypes AAV5 and AAV9 were selected for investigation in this study because in previous studies they have been characterised to exhibit the greatest propensity astrocytes in various CNS regions including spinal cord by various constitutive promoters or glial-specific promoters [6–11]. We further included the engineered serotype AAVRec2. This serotype was created within our lab by shuffling capsid sequences of the non-human primate AAV serotypes cy5, rh20 and rh39, with AAV8 [12]. These serotypes were shown in the mammalian brain to have superior transduction qualities compared with other serotypes tested in terms of transduction efficiency [5]. Therefore, we hypothesised that an AAVRec2 vector reporter gene expression controlled by a GFAP promoter would exhibit highly efficient astrocyte transduction.

An additional truncated GFAP promoter, GfaABC_1_D, was also used in this study. The GfaABC_1_D promoter is a compact GFAP promoter with the size of 694 bp. It was derived from the conventional 2.2 kb human GFAP promoter [9] by deleting 5′ nucleotides −2163 to −1758 and an internal segment from −1255 to −133. GfaABC_1_D previously displayed expression properties in transgenic mice indistinguishable from the 2.2 kb promoter [10]. This promoter allows for greater flexibility in creating therapeutic AAV constructs with less transgene size restrictions, which can be a drawback of using AAV for gene delivery [13]. Another thing to note is that a major drawback of using GFP or yellow fluorescent protein (YFP) reporter proteins is that they have a long half-life, which can lead to the accumulation of the protein within cells and can be toxic to cells. However, there are destabilised variants such as destabilised YFP (dYFP) that have been created to address this issue.

Therefore, in this present study, we investigated the transduction properties and tropism of AAV5, AAV9 and AAVRec2 using GFAP and GfaABC_1_D promoters driving the expression of dYFP using primary cell culture, spinal cord slice culture and in a rat spinal cord contusion model.

## Materials and methods

### Plasmids

This study used AAV expression plasmids expressing dYFP under control of a 2.2 kb GFAP promoter or a truncated 681-bp GFAP promoter (GfaABC_1_D) [14]. The dYFP transgene was followed by a woodchuck hepatitis virus post-transcriptional regulatory element (WPRE) and bovine growth hormone polyadenylation signal (BGHpA). The construct was flanked by AAV2 inverted terminal repeats. Therefore, the following expression cassettes were generated in this study: pAM/GFAP-dYFP-WPRE-BGHpA and pAM/GfaABC_1_D-dYFP-WPRE-BGHpA. DNA sequences were confirmed by restriction enzyme analysis and DNA sequencing (Massey Genome Service; Massey University, New Zealand).

### AAV vector packaging

Expression cassettes were packaged into the AAV serotypes: AAV5, AAV9 and Rec2 and purified as previously described [7, 15]. Genomic titres of the vectors were determined using genomic titering as previously described [16] with primers designed to WPRE and titres were matched so that equal copy numbers of each vector was used.

### Isolation and culture of primary rat spinal cord astrocytes

Primary spinal cord astrocytes were isolated from Sprague–Dawley rat pup postnatal days 0–2 (P0–2; Vernon Jansen Unit, The University of Auckland) using procedures in compliance with the University of Auckland Animal Ethics Committee Guidelines and the New Zealand Animal Welfare Act 1999. The spinal cords of two pups were excised and astrocytes were harvested as previously described [17]. Cultures were maintained in a T75 flask for at least 4 weeks with media changes every 3–4 days before plating for experimentation. For experiments, 4 × 10^4^ cells were plated in a 96-well plate (Falcon). Two days after plating, the astrocytes were transduced with the AAV vectors: AAV(5, 9 or Rec2)-GFAP-dYFP, AAV5-GfaABC_1_D-dYFP at doses of 1 × 10^9^ viral genomes (vg), 2 × 10^9^ vg and 4 × 10^9^ vg. Cultures were maintained for a further 7 days to allow for transgene expression and then fixed with 4% paraformaldehyde (PFA)-phosphate-buffered saline (PBS) for 10 min and then washed three times with PBS. Cultures were repeated a further four times (*n* = 4 biological repeats; eight pups used), including three technical repeats within each experiment).

### Fluorescent immunocytochemistry and imaging

Fixed primary spinal cord astrocytes were permeabilised using PBS + 0.1% Triton X-100 (Sigma; PBST) and then incubated overnight at room temperature in primary antibodies diluted in PBST (0.1% Triton X-100) + 4% goat serum: anti-dYFP (1:20,000; Abcam #AB290), anti-GFAP-Cy3 conjugate (1:500; Sigma #C9205). After two washes, the cells were then incubated with fluorescence-secondary antibodies at room temperature for 2 h: anti-rabbit AlexaFluor488 (1:500; ThermoFisher #ab1500077). The nuclei of cells were counterstained using Hoechst 33342 (ThermoFisher Scientific #62249). Fluorescently labelled astrocytes were imaged at ×10 magnification (three images per well; triplicate wells; four independent experiments; 36 images per group) using a Nikon Eclipse TE2000-U microscope. Individual colour channels were merged using ImageJ. Every dYFP-positive and GFAP-positive cells were counted using the cell counter plugin. As a measure of transgene expression, the channels were split, thresholded to a constant value and integrated density was measured. This value was divided by the number of dYFP-positive cells in that image as a measure of pixel intensity per cell.

### Ex vivo spinal cord slice culture

An organotypic ex vivo spinal cord slice culture system was utilised to investigate differences in the transduction efficiency and astrocyte specificity between the AAV serotypes. The method used was modified from Pakan et al. and Kearns et al. [18, 19]. Sprague–Dawley rats (10–15 days postnatal) were euthanized (two pups per culture) and the spinal cords were carefully removed and embedded in liquid 4% agar and placed on ice to set. The block was cut into 400 µm thick sections using a McIlwain Tissue Chopper (Ted Pella, Inc.) and transferred to a six-well plate transwell polyester membrane cell culture insert (Corning, Sigma). One millilitre of media was added per well (Dulbecco’s modified Eagle’s medium supplemented with 10% foetal bovine serum, penicillin–streptomycin–neomycin, 1× Fungizone (amphotericin B) and 1× Glutamax). Slices were maintained for 1 week before experimentation to allow for the slices to stabilise; ‘thinning’ caused by the death of cells reduces the thickness of the slices to approximately 150 µm. AAV(5, 9 or Rec2)-GFAP-dYFP, AAV5-GfaABC_1_D-dYFP concentrated viral vector stocks were diluted to 1 × 10^11^ vg/mL in 1× PBS-MK, 0.001% pluronic acid. Slices were transduced with 1 × 10^9^ vg of viral vectors by pipetting the vector drop-wise onto the surface of the slice. Slices were returned to the incubator and maintained for 4 days before the slices were washed with PBS and fresh media were replaced. Slices were maintained for a further 3 days at which point they were fixed for 2 h in 4% PFA-PBS at 4 °C. The slices were then washed three times to remove the PFA and stored in PBS + 0.1% sodium azide at 4 °C until required for immunohistochemistry. Slice cultures were repeated a further four times (*n* = 4 biological repeats; eight pups used), including three technical repeats within each experiment).

### Fluorescent immunohistochemistry on spinal cord slice cultures

Following blocking in PBST + 4% goat serum for 2 h, the slices were incubated overnight at room temperature in primary antibody diluted in PBST + 4% goat serum: anti-dYFP (1:20,000; Abcam #AB290), anti-GFAP-Cy3 conjugate (1:500; Sigma #C9205). After 2 × 10-min washes, the cells were then incubated with fluorescence secondary antibodies at room temperature for 4 h: anti-rabbit AlexaFluor488 (1:500; ThermoFisher #ab1500077). This was followed by a further 2 × 5-min washes and the slices were mounted with Prolong Gold.

### Imaging and analysis of fluorescently labelled spinal cord slices

Fluorescently labelled spinal cord slices were imaged using an EVOS FL Auto microscope (ThermoFisher Scientific). Mosaic images of each colour channel were taken at ×10 magnification automatically using the microscope software; ×20 magnification images were captured for cell counting. Images were analysed using ImageJ software by an independent and masked observer. As a measure of astrocyte-specific transduction, every dYFP-positive cell was counted in the green (FITC filter) channel using the cell counter plugin. The channel was then switched to the red (GFAP filter) channel and every dYFP plus GFAP-positive cell was counted and presented as a proportion of astrocytic dYFP-positive cells.

### Spinal cord contusion injury and AAV vector delivery

Animals were obtained from the Vernon Jansen Unit, the University of Auckland in compliance with the University of Auckland Animal Ethics Committee Guidelines and the New Zealand Animal Welfare Act 1999. To determine the number of animals required for behavioural assessment a power calculation was carried out (http://hedwig.mgh.harvard.edu/sample_size/size.html). To see a difference in Basso, Beattie and Bresnahan (BBB) score of 2 points (as in our previous studies looking at connexin regulation for SCI [20]), assuming a standard deviation of the BBB scores between the treatment and control group of 1 and using a significance level of 0.05 and a power of 0.8 it was determined that 12 animals per group would be required. Animals were housed in a 12:12 light/dark cycle both prior and following surgery. Animals were operated on in order from heaviest to lightest and were assigned to experimental groups in alternation. For the thoracic contusion, a laminectomy was performed to expose the spinal cord at spinal level T10 (*n* = 12 per group). The animal was then transferred to an Infinite Horizon Impactor (Precision Systems Instrumentation) and impacted in the midline with a force of 175 KDyne. Immediately after contusion injury, the rats were transferred to a stereotaxic frame (David Kopf Instruments). Local anaesthesia bupivacaine (1 mg/kg) was placed on the exposed spinal cord and allowed to absorb for 5 min. A glass micropipette with a tip diameter of 100 µm coupled to 10 µL Hamilton syringe and a Micro Syringe Pump Controller (World Precision Instruments) was used to inject viral vectors into the spinal cord (vectors: AAV5-GFAP-dYFP and AAV5-GfaABC_1_D-dYFP were tested). One injection of 4 × 10^9^ vg (1 µL) at a depth of 1 mm was made at each corner of the lesion site (four in total) at a rate of 200 nL/min. The needle was left in the spinal cord for a further 2 min before it was removed. The subcutaneous tissue was sutured using an absorbable suture (ChomicGut) and the skin was sutured closed suing nylon suture (Ethicon, Johnson and Johnson).

### Behavioural assessment

The BBB locomotor rating scale was used to measure motor function after spinal cord thoracic contusion. Testing was carried out in a circular open field (100 cm diameter with 20 cm walls). Animals were acclimatised to the behavioural testing apparatus for 1 week prior to the surgery. Following the surgery, rats were tested weekly for 4 weeks in the open field for 5 min each time. This length of time has been determined as providing sufficient time to observe and record behavioural recovery of individual rats with minimal risk of missing key findings. The scoring was carried out by blinded observers as previously described by Basso et al. where motor functions are ranked from 0 (total paralysis) to 21 (normal movement) [21].

### Tissue processing and DAB immunohistochemistry

Animals were overdosed with sodium pentobarbitone (Pentobarb 300; 100 mg/kg, i.p) and transcardially perfused with 0.9% saline followed by 4% PFA in 0.1 M phosphate buffer. Immediately after perfusion, the whole spinal cord of the animal was removed. The cord was postfixed in 4% PFA in 0.1 M phosphate buffer overnight at 4 °C before being cryoprotected in 20% sucrose in 0.1 M PB, followed by 30% sucrose in 0.1 M. The tissue was embedded in Optimal Cutting Temperature (OCT) compound and 1 cm of the cord with the lesion located centrally was cut transversely. In all, 30-μm thick serial sections were cut using a cryostat and stored in PBS + 0.1% sodium azide at 4 °C until required. Diaminobenzidine (DAB) immunohistochemistry was used to visualise dYFP gene expression in the spinal cord sections. Spinal cord sections were mounted onto a series of positively charged slides, such that the adjacent sections mounted on each slide represented one section within a 600 μm block. Endogenous peroxidase activity was quenched with 1% hydrogen peroxide in 50% methanol for 20 min. Sections were incubated with rabbit anti-GFP primary antibody (reactivity to dYFP; Abcam AB290) diluted 1:50,000 in PBST + 4% goat serum overnight at room temperature Followed by biotinylated goat-anti-rabbit secondary antibody diluted 1:250 in PBST + 4% goat serum for 2 h. The sections were then incubated with ExtrAvidin Peroxidase (Sigma) diluted 1:250 in PBST + 4% goat serum for 2 h. The sections were then stained with DAB; 0.2 mg/mL DAB, 0.01% hydrogen peroxide, 0.1 M PB pH 7.2. Mounted slides were imaged using a Leica DMR microscope at ×2.5 magnification using Nikon NIS Elements for image acquisition. As a measure of viral vector gene expression level, the percentage fraction of staining was calculated using ImageJ software. Each image was converted to 8 bit and thresholded using identical thresholding values to report transgene staining. Tissue from animals that did not receive vector infusion was used as a negative control to confirm positive staining. The total area of the stained section was expressed as a percentage fraction of the total section area. Sections from every animal used in the study were stained.

### Immunofluorescence and confocal imaging

Fluorescent immunohistochemistry was used to visualise viral vector gene expression in which cells the gene expression was occurring. As determined by DAB immunohistochemistry, sections were selected that had highest vector expression in the series. The sections were blocked and permeabilised with 10% goat serum in PBST (0.2%). Sections were incubated with primary antibodies; rabbit-anti-dYFP 1:20,000 (Abcam #ab290), mouse-anti-dYFP 1:200 (Abcam #ab291), mouse-anti-GFAP-Cy3 1:1000 (Sigma #C9205), mouse-anti-NeuN 1:250 (Merck #MAB377), rabbit-anti-Iba1 1:250 (Wako #019–19741), rabbit-anti-olig2 diluted 1:500 in PBST + 4% goat serum overnight at room temperature and then incubated with complementary secondary antibodies; all AlexaFluor diluted 1:750 in PBST + 4% goat serum for 4 h at room temperature. Sections were mounted onto positively charged glass slides and coverslipped with prolong gold. After 2 days of curing, the slides were imaged on an Olympus FV1000 confocal laser scanning microscope at ×60 magnification. Sections from every animal used in the study were stained.

### Data exclusions and statistical analyses

Animals were excluded from the study if their combined hindlimb BBB score was >5 at day 1 after spinal cord surgery; if their BBB scores did not improve past a combined score of 8 by the end of the 4 weeks, and if the animal was euthanized because of complications of the surgery. This resulted in *n* = 9 for both AAV5-GFAP-dYFP and AAV5-GfaABC_1_D-dYFP groups. Values are expressed as the mean ± standard error of the mean (SEM). Statistical analyses were conducted using Prism version 7.0 (GraphPad, La Jolla, USA). One-way analysis of variance (ANOVA) followed by a Tukey’s multiple comparisons was used to determine statistically significant differences between the means of groups split by one independent variable (treatment) where *P < 0.05, **P < 0.001, ***P < 0.0001. Two-way ANOVA’s and a Bonferroni’s multiple comparisons test were used to compare the mean differences in data between groups that have been split by two independent variables (e.g., treatment and time) where *P < 0.05, **P < 0.001, ***P < 0.0001.

## Results

### In vitro transduction efficiencies and transgene expression properties of the AAV serotypes: 5, 9 and REC2

The transduction efficiencies and transgene expression levels were investigated in primary cell culture to determine the most effective serotype for transducing spinal cord-derived astrocytes. Cultures isolated from postnatal rat spinal cord were found to be rich in GFAP-positive astrocytes with minor populations of oligodendrocytes and microglia (Supplementary Fig. 1). To determine transduction efficiencies and expression levels, cultures were transduced by the AAV5, AAV9 or AAVRec2 containing a dYFP transgene under the control of a GFAP promoter. Following early preliminary results that showed AAV5 as the most efficient vector compared with AAV9 and AAVRec2, an additional AAV5 vector containing the truncated 681 bp GfaABC_1_D promoter was included in the study. AAV vectors were applied to the cultures at either 1 × 10^9^, 2 × 10^9^ or 4 × 10^9^ vg and cells were fixed 7 days later. Immunocytochemistry was used to visualise reporter gene and GFAP expression.

**Fig. 1:**
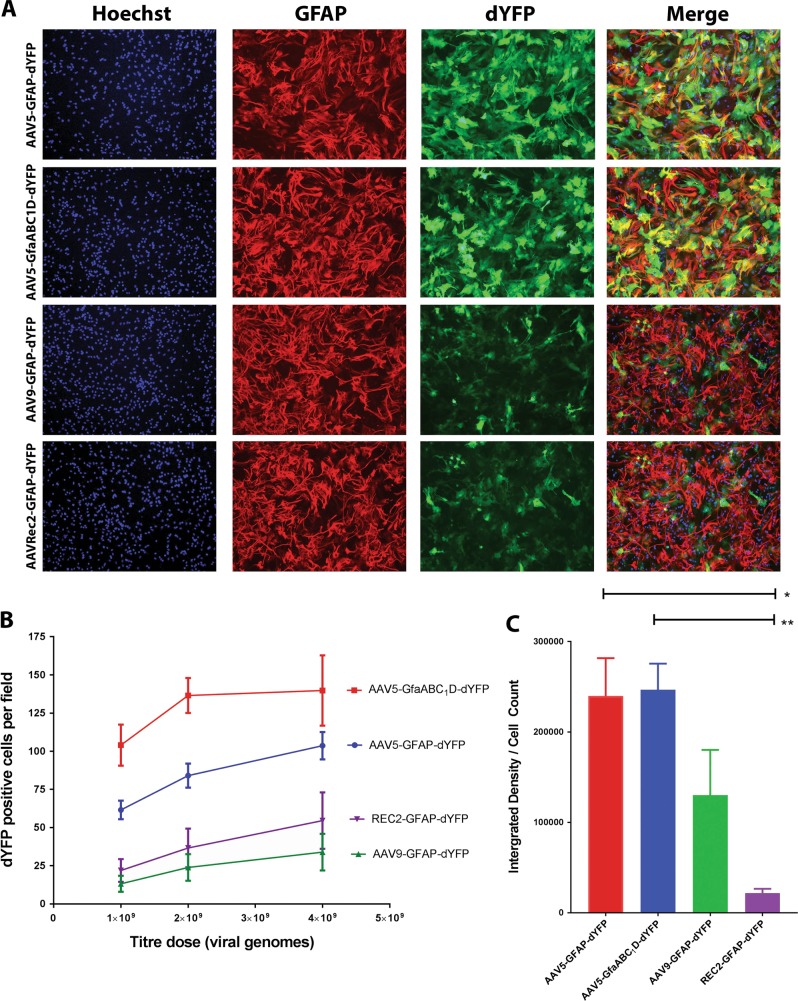
The AAV5 serotype leads to the most efficient transduction and strongest transgene expression in primary spinal cord astrocytes. **a** Vector constructs containing a dYFP transgene under the control of a GFAP or GfaABC_1_D promoter were packaged into AAV serotypes (5, 9 or Rec2) and transduced cultures; AAV5-GFAP-dYFP, AAV5-GfaABC_1_D-dYFP, AAV9-GFAP-dYFP, AAVRec2-GFAP-dYFP (4 × 10^9^ vg/well). dYFP gene expression was visualised using fluorescent immunocytochemistry and captured at ×10 magnification using a Nikon Eclipse TE2000-U microscope. Each is a representative image. Scale bar = 200 µm. **b** The number of dYFP-positive cells per image were counted. Values represent the mean and standard error of the mean (*n* = 4; independent cultures). Two-way ANOVA followed by Tukey’s multiple comparisons were used to determine statistical significance (not reported on the graph). **c** dYFP gene expression was visualised using fluorescent immunocytochemistry and images captured at ×10 magnification using a Nikon Eclipse TE2000-U microscope using identical settings and the number of dYFP-positive cells were counted. The integrated densities were determined for each image using ImageJ and used to determine the fluorescent intensity per cell. Values represent the mean and standard error of the mean (*n* = 4 independent cultures; three technical repeats for each; three images per technical repeat). One-way ANOVA followed by Tukey’s post-hoc analysis *P < 0.05, **P < 0.001

At the highest viral dose (4 × 10^9^ vg), the AAV9-GFAP-dYFP vector mediated the least efficient transduction as determined by the mean number of dYFP-positive cells (33.89 cells/10× objective field) (Fig. 1a, b). AAVRec2-GFAP-dYFP was slightly more efficient than AAV9 (54.54 cells/field). The AAV5 serotype (with either promoter) showed the greatest efficiency of transduction (*P* < 0.02) with the AAV5 serotypes with the truncated-GfaABC_1_D promoter showing the greatest number of dYFP-positive cells (Fig. 1b). At the highest viral dose of 4 × 10^9^ vg, there was an average of 139 dYFP-positive cells per 10× objective field. In comparison, there were 103 positive cells for the full GFAP promoter, although this was not statistically different (*P* = 0.317).

Transgene expression levels were determined by dividing the integrated density of thresholded images by the cell count of each image, giving an indication of the average fluorescence intensity of individual cells (Fig. 1c). AAVRec2-GFAP-dYFP showed the lowest level of transgene expression, significantly less than AAV5-GfaABC_1_D-dYFP and AAV5-GFAP-dYFP (*P* < 0.011). AAV9-GFAP-dYFP showed a level of expression that was at a level between the other vectors tested. Though the AAV5-GfaABC_1_D-dYFP vector showed greater transduction efficiency it did not also show greater transgene expression compared with AAV5-GFAP-dYFP, with no difference in transgene expression (*P* = 0.99) suggesting similar levels of transcriptional activity between the GFAP and GfaABC_1_D promoters.

### Transgene expression of AAV serotypes 5, 9 and Rec2 in ex vivo spinal cord slice cultures

Organotypic slice cultures allow for simplistic gene transfer and visualisation while retaining 3D cytoarchitecture and a heterogeneous population of cells. We therefore employed spinal cord slice culture to investigate the astrocyte specificity of each AAV vector.

**Fig. 2:**
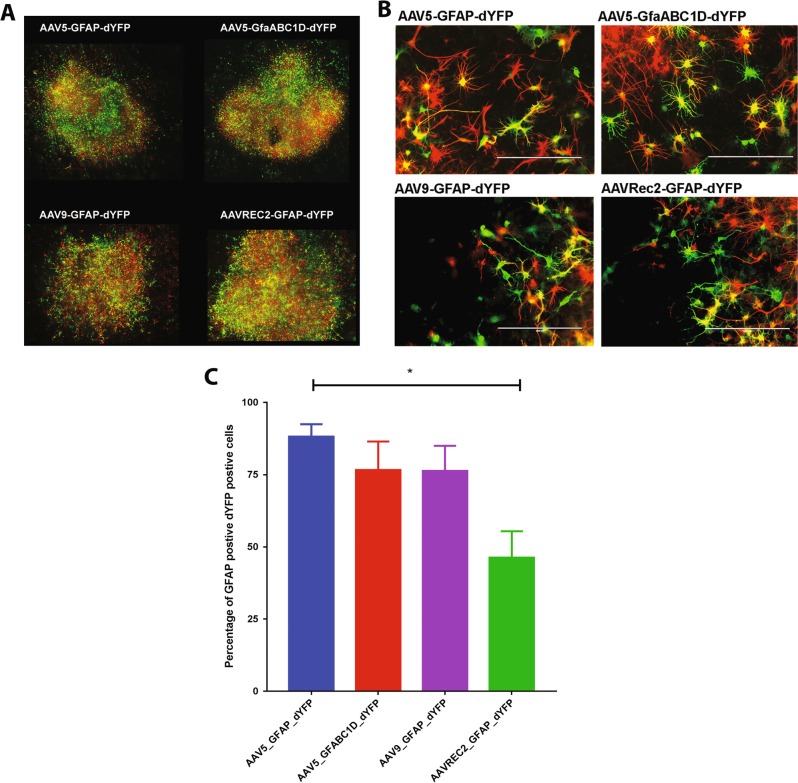
Organotypic slice cultures transduced with AAV vectors show robust gene expression and AAV5 results in the greatest astrocyte-tropic transductions. Spinal cord were excised from P10 to 15 rat pups, sliced and then cultured. Slices were transduced with AAV vectors: AAV5-GFAP-dYFP, AAV5-GfaABC_1_D-dYFP, AAV9-GFAP-dYFP, and AAVRec2-GFAP-dYFP (1 × 10^9^ viral genomes each). Gene expression was developed for 7 days before fixing. Immunohistochemistry was used to visualised dYFP gene expression (green) and GFAP expression (red). **a** ×10 magnification images were stitched together to display the whole slice. **b** Images were captured on an EVOS FL Auto imaging station at ×20 magnification to analyse colocalisation of dYFP and GFAP. Images are representative images. **c** Data are represented as percentage of GFAP-positive dYFP-positive cell ± S.E.M. (*n* = 4 independent cultures; three technical repeats for each; three images per technical repeat) independent slice cultures. A one-way ANOVA with Tukey’s multiple comparison test was used to determine statistical differences between the vectors, *P < 0.05

Application of AAV5-GFAP-dYFP, AAV5-GfaABC_1_D-dYFP, AAV9-GFAP-dYFP and AAVRec2-GFAP-dYFP (1 × 10^9^ vg per slice) all resulted in strong dYFP transgene expression (Fig. 2a). Immunohistochemistry was performed to co-label GFAP and dYFP expression to determine whether transgene expression was occurring in GFAP-positive astrocytes (Fig. 2b). The AAV5 serotype containing the full GFAP promoter exhibited the mean highest GFAP/dYFP colocalisation; 88% of dYFP-positive cells were also GFAP-positive astrocytes. The AAV5 serotypes containing the truncated GfaABC_1_D promoter resulted in less dYFP/GFAP colocalisation with 76% of dYFP-positive cells being also GFAP-positive (Fig. 2c). This percentage was matched by the AAV9-GFAP-dYFP vector. There was, however, some variability in these results and the values between these three vectors were not statistically different from each other. The lowest amount of dYFP transgene expression within GFAP-positive astrocytes was observed for the AAVRec2-GFAP-dYFP vector where there was only 46% of double-positive cells; this was significantly different to the AAV5-GFAP-dYFP group (*P* = 0.022).

### Analysis of dYFP expression spread and motor function in an in vivo rodent SCI model

Based on the data obtained from the in vitro and ex vivo investigation, we evaluated the transduction efficiencies between the AAV5-GFAP and the GfaABC_1_D promoter vectors in an in vivo rodent model of spinal cord contusion. Immediately after a 175 KDyne contusive injury, four injections of the vector were delivered: one at each corner of the injury site and at a depth 1.5 mm. Immunohistochemistry was used to investigate the spread of the dYFP transgene.

Initially, it was intended that dYFP-positive cells would be counted per spinal cord section and results would then be expressed as a number of dYFP-positive cells per area. However, because dYFP transgene expression results in differing intensities and coupled with the undulating structural arrangement of astrocytes, it was not possible to accurately count how many individual cells are expressing the transgene (Supplementary Fig. 2A, B). Instead, images were captured using identical microscope settings and images were thresholded to the DAB staining. The percentage of the total area of the spinal cord stained was used to compare the transduction area and spread between the two vectors investigated (Supplementary Fig. 2C, D).

**Fig. 3:**
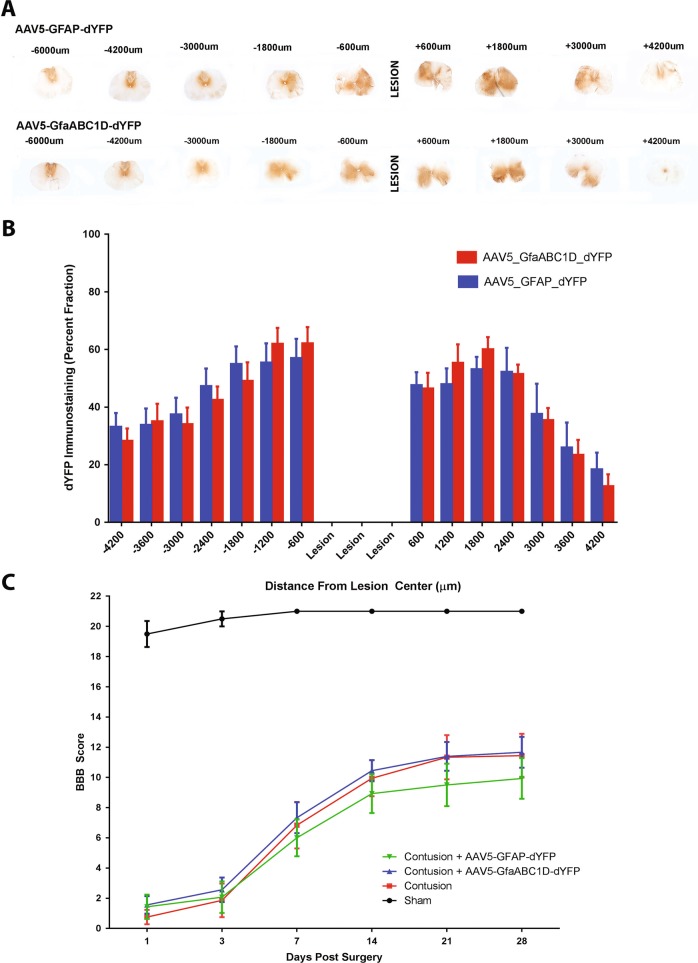
High transgene expression by GFAP-dYFP or AAV5-GfaABC_1_D is observed through >12 mm of spinal cord tissue and does not worsen locomotor scores. Rats received 175 KDyne contusive injuries and then immediately received four injections of either AAV5-GFAP-dYFP or AAV5-GfaABC_1_D (4 × 10^9^ vg at each). Four weeks later, the animals were euthanized and DAB immunohistochemistry was performed to visualise dYFP transgene expression in the spinal cord tissue. **a** Images were captured at ×2.5 magnification on a Leica DMR upright microscope. **b** Images were thresholded and ImageJ was used to determine the percentage of tissue that was immunoreactive for transgene expression. Each bar represents the mean ± SEM of transgene expression within 600 µm blocks from the injury centre (*n* = 9). **c** The BBB open field locomotor scale was used to investigate whether the injections had an impact on their locomotion. Data represent the mean ± SEM (sham *n* = 4; *n* = 9 for the other groups). A two-way repeated-measure ANOVA followed by a Bonferroni’s multiple comparison was used to determine if there was any statistical difference between contusion groups

Both vectors showed robust gene expression in spinal cord tissue (Fig. 3a). We found that the two vectors showed equal transduction area and transduction spread. From the penumbra of the lesion site to 600 µm rostral, on average approximately 60% of the spinal cord sections were positive for dYFP immunostaining (Fig. 3b). Expression caudal to the injury tapered off more quickly than the rostral side of the injury; at 4.2 mm from the lesion centre expression was <20% of the spinal cord section whereas at 4.2 mm rostral there was still approximately 30% expression by either vector. Taking into account that the lesion covers 1.8 mm of tissue, in total, gene expression was spread through over 12 mm of tissue for both of the vectors.

In order to determine if vector injection may lead to damage of the cord, the BBB open field test was used. This is a sensitive and reliable rating scale for open field testing of SCI rats. Rats were assessed for 4 weeks following a 175 KDyne contusive injury and four injections of either AAV5-GFAP-dYFP or AAV5-GfaABC_1_D-dYFP. Sham surgery animals had a decrease in BBB score by 2 points 1 day post injury (Fig. 3c). By day 7, this had returned to a complete score of 21. One day after the injury, all injury groups had a BBB score of less than 2; slight movement of one or two joints (Fig. 3c). This confirms that all animals received a consistent impaction. Injury groups show improvements in the BBB scores from day 1 through to week 3 at which point this improvement plateaued, which is expected from this moderate contusive injury. Animals that received AAV5-GfaABC_1_D-dYFP injections had a BBB profile that overlapped with the contusion only control, implying that these injections do not worsen the injury. Animals that received AAV5-GFAP-dYFP injections had a BBB profile that was consistently lower than that of the contusion control and the contusion + AAV5-GfaABC_1_D-dYFP groups, however, there was no significant difference between any of the injury groups.

### Qualitative analysis of cell type specificity of AAV5-GFAP-dYFP and AAV5-GfaABC_1_D in the contused spinal cord

Spinal cord sections at the point of highest transgene expression (600 µm rostral to the lesion) were selected and immunohistochemistry using cell-specific markers was performed to determine the cell types in which the transgene was being expressed. Qualitative evaluation was performed using a scoring system adapted from Cearley et al. and Petrosyan et al. [11, 22].

**Fig. 4:**
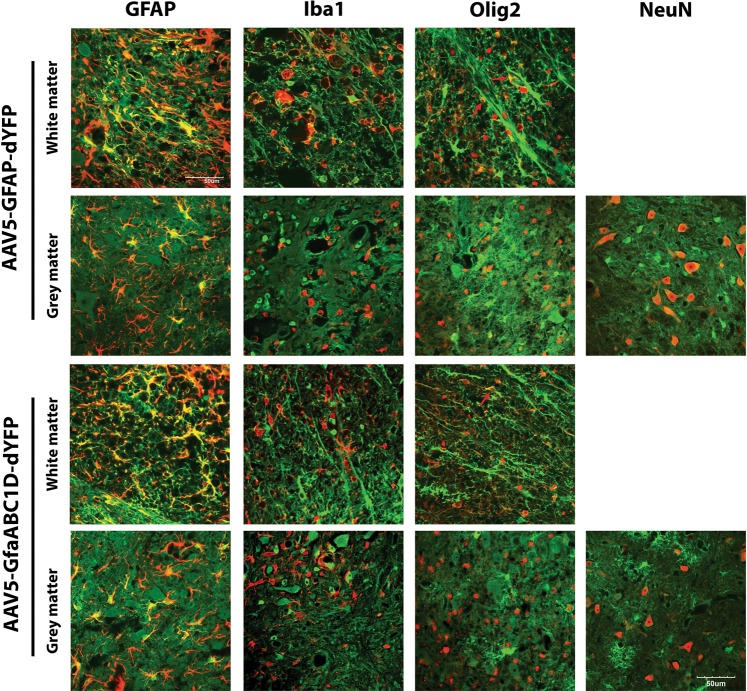
AAV5 vector dYFP expression driven by GFAP or GfaABC_1_D promoters results in astrocyte-specific transgene expression. Constructs containing a dYFP reporter transgene driven by either a GFAP or GfaABC_1_D promoter were packaged into AAV5 vectors and four infusions of 4 × 10^9^ viral genomes into the contused rodent spinal cord. Tissue was processed for immunohistochemistry to detect dYFP (green) colocalisation with the cell type markers: GFAP, Iba1, Olig2 and NeuN (red). Images were captured at ×60 magnification on an Olympus FV1000 confocal microscope (*n* = 9). Images presented are representative images. Scale bar = 50 µm

Transgene expression using both AAV5-GFAP-dYFP and AAV5-GfaABC_1_D-dYFP to mediate gene delivery was largely astrocytic (Fig. 4). Immediately rostral to the injection site (from the penumbra of the lesion site to 600 µm rostral), there is near saturation of transduced GFAP-positive astrocytes (Fig. 4, Table 1) Transgene expression in neurons, as indicated by colocalisation of dYFP and NeuN immunoreactivity, was present in spinal cords transduced with either AAV5-GFAP-dYFP or AAV5-GfaABC_1_D-dYFP. Large motor neurons within the ventral horn showed the strongest level of neuronal transgene expression (Supplementary Fig. 3). Neurons in areas of the grey matter other than the dorsal or ventral horn showed only low levels of dYFP expression (Supplementary Fig. 3). An antibody against Iba-1 was used to visualise microglia and blood-derived macrophages. Extensive staining of activated microglia and foamy macrophages were observed due to the proximity of the lesion centre, however, no transgene expression was observed in these cells. An antibody against oligodendrocyte transcription factor 2 (Olig2) was used to identify oligodendrocytes in the spinal cord tissue. In tissue transduced by either AAV5-GFAP-dYFP or AAV5-GfaABC_1_D-dYFP very weak transgene fluorescence was observed in very few Olig2-positive cells or processes (Fig. 4; arrows).

**Table 1:** Scores for AAV tropism of astrocytes, neurons, microglia/macrophages and oligodendrocytes

AAV vector	Astrocytes (GFAP)	Neurons (NeuN)	Microglia/macrophage (Iba1)	Oligodendrocytes (Olig2)
AAV5-GFAP-dYFP	++++	++	–	+
AAV5-GfaABC_1_D-dYFP	++++	++	–	+

Scoring: (–) no observable transgene positive cells; (+) very few positive cells or processes; (++) several positive cells or processes per image; (+++) many positive cells or processes per image; (++++) robust transduction region completely saturated with positive cell or processes (adapted from Petrosyan et al.) [11]

## Discussion

It is becoming increasingly clear that astrocytes play a critical role in SCI pathogenesis, as well as other neurotrauma or neurodegenerative conditions. Therefore, AAV vectors that exhibit a propensity toward transducing astroglial cell populations are attractive candidates for the development of targeted treatments.

In this study, we investigated the transduction efficiency, transgene expression and cellular tropism of the AAV serotypes: AAV5, AAV9 and AAVRec2 in spinal cord astrocytes using in vitro, ex vivo and in vivo models. AAV5 and AAV9 were selected for investigation in this study because previously they were characterised to exhibit the greatest propensity for transducing astrocytes in various CNS regions, including the spinal cord [6–11]. AAVRec2 is an engineered hybrid serotype that is related to AAV8. It was created by shuffling the capsid sequences of three non-human primate AAV serotypes: cy5, rh20 and rh39, with AAV8—these serotypes were shown in the brain of non-human primates to have superior transduction qualities when injected into the hippocampus—albeit the cy5, rh20 and rh39 were largely neuron centric [5]. As AAV8 showed tropism to target astrocytes, we therefore hypothesised that the engineered AAVRec2 would exhibit greater transduction and astrocyte tropism than its AAV8 predecessor. At the commencement of this study, there was no literature pertaining to the transduction properties of the AAVRec2 vector in the brain or spinal cord tissue. However, more recently the transduction properties of an AAVRec2 vector containing a chicken β-actin (CBA) promoter was investigated in the mouse thoracic spinal cord in comparison with the AAV9, AAVRec3 and AAVRec4 serotypes [4]. AAVRec2 resulted in approximately half the transduction range (longitudinal transduction spread) compared with the other three serotypes, however, transgene expression levels as measured by GFP fluorescent intensity were comparable [4]. Although gene expression was largely neuronal, AAVRec2 showed a greater number of transduced astrocytes compared with AAV9 indicating that this could be improved through the use of an astrocytic promoter such as GFAP. In our primary cell culture experiments, the AAV5 serotype showed superior transduction efficiency and transgene expression at all three vector doses compared with AAV9 and AAVRec2. Contrary to the literature, AAV9-GFAP-dYFP resulted in fewer transduced cells while showing greater transgene expression compared with AAVRec2-GFAP-dYFP [4]. Given that vector transduction properties in an isolated culture do not give an indication of cellular tropism, the vectors were subsequently tested in rat spinal cord slice ex vivo culture. Interestingly, spinal cord slice cultures showed robust transgene expression for all of the serotypes tested. This is possibly due to dosing the slices with a large number of viral particles that may have saturated each slice. AAV5 showed the highest degree of astrocyte transduction (AAV5-GFAP = 88%; AAV5-GfaABC_1_D = 77%) as indicated by the percentage of transgene-expressing cells that were GFAP-positive astrocytes. AAV9 reported approximately 75% of colocalisation while AAVRec2 was the least astrocyte-specific with <50% colocalisation of dYFP and GFAP.

When combined with constitutively active promoters such as cytomegalovirus (CMV) or CBA, exclusive neuronal transduction by AAV5, AAV9 and AAV8 has been observed in various CNS regions, as well as in the spinal cord. In a previous study, using a CMV promoter, AAV9 was shown to be a far more effective serotype in terms of transgene expression compared with AAV5, while having comparable transduction efficiency [11]—a result that is not consistent with our in vitro and ex vivo studies. Conversely, using a CBA promoter, AAV5 displayed more than double the number of GFP-positive neurons and twice the transduction spread compared with AAV9 when infused into the rat spinal cord—a result that is consistent with our results [23]. The AAV5 serotype has been previously shown to exhibit almost completely astrocyte-specific transduction (99%) when a GFAP promoter was used after infusion into the mouse hippocampus [9]. This greater astrocyte specificity could be explained by the fact that AAV5 is the most divergent of the serotypes and shares approximately only ~55% sequence homology with other serotypes [24], and for this reason, AAV5 may exhibit unique transduction properties. Additionally beneficial is that AAV5 appears to be immunologically distinct from other serotypes and has minimal cross-reactivity against pre-existing wild-type AAV2 neutralising antibodies [25]. The greatly reduced transgene expression within GFAP-positive astrocytes by AAVRec2 may be explained by the fact the cy5, rh20 and rh39 serotypes from which the vector was engineered are all largely neuron centric and this may not be altered by the combination with AAV8 and a GFAP promoter. Furthermore, this observation might be due to heterogeneity in astrocyte populations as well, i.e., astrocytes in one brain region differ to another [2]. It must be noted, however, that results pertaining to AAV tropisms and transduction efficiencies in the CNS are often difficult to interpret due to differences in vector titres, purification methods, route of administration, DNA regulatory elements and transgenes, duration of transgene expression and animal species used [26].

The ~4.7 kb packaging capacity of AAVs imposes a sequence length restriction on the therapeutic genes that can be cloned into the expression cassette. Larger expression plasmids compromise the stability of the vector capsids leading to inefficient assembly and transduction [27, 28]. Although the 2.2 kb GFAP promoter effectively mediates transgene expression in astrocytes, it is a relatively large promoter that occupies almost half of the packaging capacity. Given that it is desirable to use smaller promoters to ease length restriction on therapeutic sequences, we tested the ability of the recently characterised shorter GFAP promoter GfaABC_1_D, which lacks redundant C-regions (for full details refer to Lee et al. [14]). After preliminary results in cell culture experiments in which the AAV5 vectors displayed greater transduction efficiency and transgene expression, an AAV5 vector containing the GfaABC_1_D was created and tested to investigate if it would retain high transduction qualities. Interestingly, AAV5-GfaABC_1_D-dYFP transduction of cell cultures led to a far greater number of dYFP-positive cells compared with the full GFAP promoter in the same serotype, while transgene expression levels were comparable.

Based on the information presented from the in vitro and ex vivo experiments the AAV5 serotype was selected for in vivo studies—based on its superior transduction efficiency, transgene expression and astrocyte tropism compared with AVV9 and AAVRec2. The AAV5 serotype with the 2.2-kbp GFAP or the 681-bp GfaABC1D promoter were investigated in an in vivo model of spinal cord contusion to determine transgene expression and cellular tropism. First, the infusions of the vectors into the spinal cord did not affect motor functions as measured by the BBB scoring system, suggesting no mechanical disruption and/or toxicity caused by the vector itself. Both vectors showed robust gene expression and equal transduction area and transduction spread. This spread was observed to be through 6 mm rostral to the injury site and 4.2 mm caudal, taking into account that the lesion covers 1.8 mm of tissue, this totals to over 12 mm of tissue expressing the transgene. In a previous study that infused AAV5 into the contused rat spinal cord using a CMV constitutively active promoter, transduction was seen through 8 mm of tissue when a comparable titre was used (1.36 × 10^10^ vg compared with 1.6 × 10^10^ vg used in this study) [11]. In this study, we saw transgene expression through over 12 mm of tissue from the injury site indicating that coupling particular capsid-tropism to particular promoters is advantageous in terms of spread.

We next investigated the cellular tropism of the vectors in the spinal cord tissue. AAV5-GFAP-dYFP and AAV5-GfaABC_1_D-dYFP were both found to be selective for astrocyte transduction in both the grey and white matter of the rat spinal cord. Neuronal transgene expression, however, was detected with both vectors. Motor neurons in the ventral horn showed relatively high transgene expression, whereas other neurons showed weak transgene expression and no dYFP was observed in neuronal cell bodies in the dorsal horn. This indicates that neurons in different regions within the spinal cord have a different repertoire of cell surface receptors or different transcriptional activity. Moreover, given that cell-specific activity of the GFAP promoter may not necessarily represent tropism of AAV serotype but rather the transcriptional activity of the promoter within that cell type. An absence of dYFP colocalisation with the microglia marker, Iba1 indicates either an inability of AAV5-GFAP-dYFP and AAV5-GfaABC_1_D-dYFP to transduce microglia and/or inactivity of the GFAP promoter in microglia. Few Olig2 cells were positive for dYFP indicating the vector shows little tropism for oligodendrocytes, however, these cells do not display a classical oligodendrocyte phenotype. Olig2 is expressed by glial precursor cells and GFAP transcription may also be occurring in these cells and so it is feasible that we are observing transgene expression in a glial precursor population [29, 30].

This study provides evidence for GFAP or the truncated GfaABC_1_D promoter-driven gene expression via an AAV5 vector as a leading AAV gene therapy candidate for the selective and wide-spread transduction of spinal cord astrocytes. This ability to alter the tropism of AAV vectors by combining suitable serotypes to cellular promoters means that it is possible to expand the potential utility of AAV vectors for the treatment of SCI where astrocytes play a critical role in the pathogenesis.

## Supplementary information

Revised supplementary information

Supplementary Figure 1

Supplementary Figure 2

Supplementary Figure 3
