# Supplementary material for: Astrocyte-selective AAV gene therapy through the endogenous GFAP promoter results in robust transduction in the rat spinal cord following injury
Source: Gene Ther. 2019 Apr 8;26(5):198–210. doi: 10.1038/s41434-019-0075-6 (PMC6760677; doi:10.1038/s41434-019-0075-6)
Supplement: Supplementary file 1 — Revised supplementary information [file 41434_2019_75_MOESM1_ESM.docx]

## Supplementary Information

Supplementary Figure 1: Characterisation of the cell types present in primary rat spinal cord cultures. Cultures derived from P0-2 rat spinal and immunocytochemistry was used to identify specific cell populations. The cultures are predominately astrocytes, identifiable by extensive immuno-reactivity to GFAP (a). A small number of MBP positive and Iba1-positive cells were present (d, e). There is no immuno-reactivity towards GFP/dYFP (c). Scale = 300 µm.

Supplementary Figure 2: The undulating nature of spinal cord astrocytes makes cell counting not possible. Rats received at 175 KDyne contusive injuries and then immediately received four injections of either AAV5-GFAP-dYFP or AAV5-GfaABC_1_D (4x10^9^ vg at each). Four weeks later the animals were euthanized and DAB immunohistochemistry was performed to visualise dYFP transgene expression in the spinal cord tissue. Images were captured at on a Leica DMR upright microscope at A) 10x magnification and B) 40x magnification. C, D) Images were captured using identical microscope setting; images were then thresholded to a consistent value to determine dYFP staining. Measurement of the total amount of staining per the whole section was then calculated.

Supplementary Figure 3: Low magnification images of thoracic spinal cord sections transduced with AAV vectors exhibit transduction of large motor neurons in the ventral horn and low expression in central grey matter. Constructs containing a dYFP reporter transgene driven by either a GFAP or GfaABC_1_D promoter were packaged into AAV5 vectors and four infusions of 4x10^9^ viral genomes into the contused rodent spinal cord. Four weeks later the animals were euthanised and tissue was processed for immunohistochemistry to detect dYFP colocalisation with NeuN fluorescence. Images were captured at 10x magnification using an Olympus FV1000 confocal microscope. The grey matter of each merged image is highlighted. Images are representative images.
