# Supplementary figures and images for: Astrocyte-selective AAV gene therapy through the endogenous GFAP promoter results in robust transduction in the rat spinal cord following injury
Source: Gene Ther. 2019 Apr 8;26(5):198–210. doi: 10.1038/s41434-019-0075-6 (PMC6760677; doi:10.1038/s41434-019-0075-6)

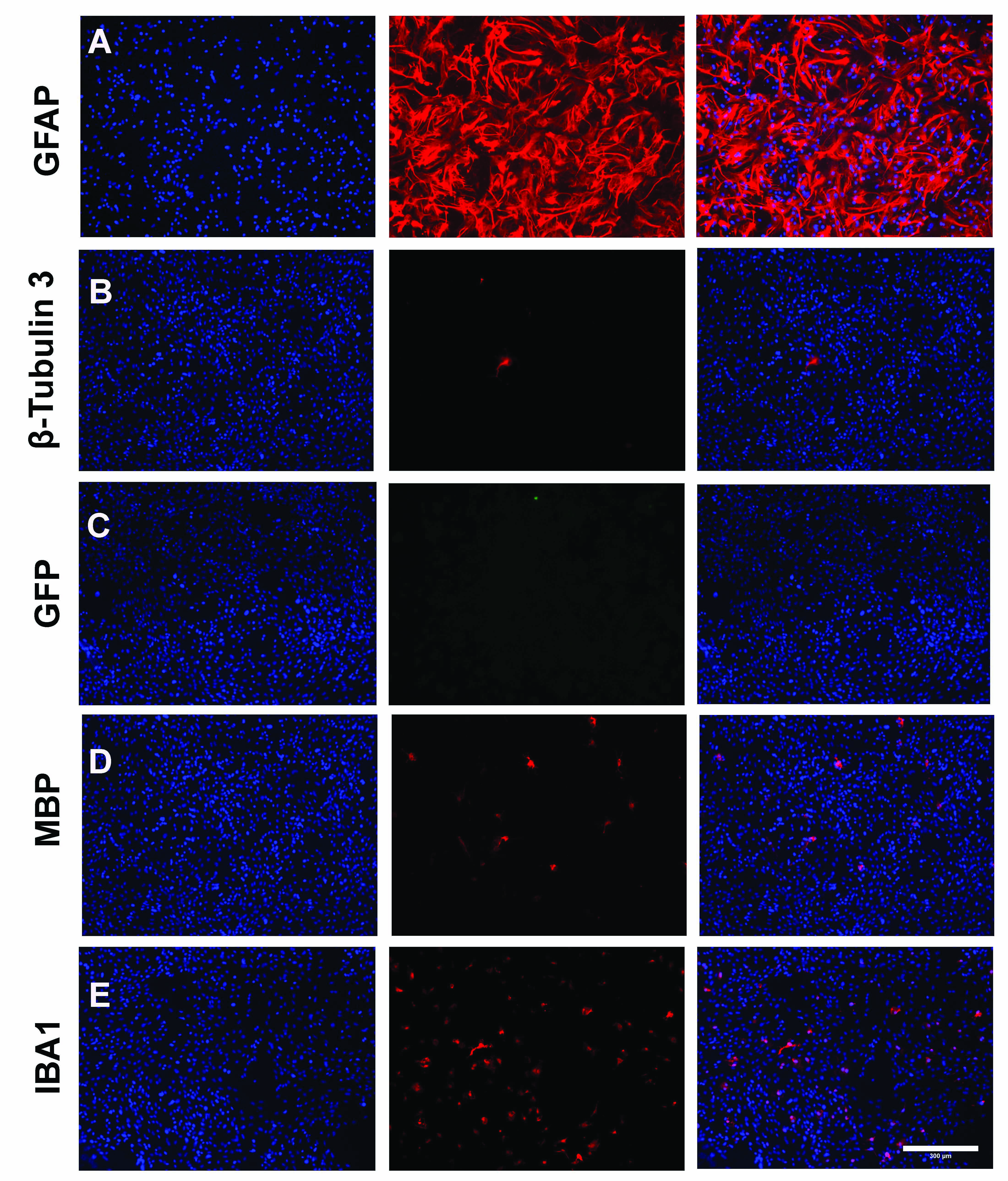

Supplement: Supplementary file 2 — Supplementary Figure 1 [file 41434_2019_75_MOESM2_ESM.jpg]

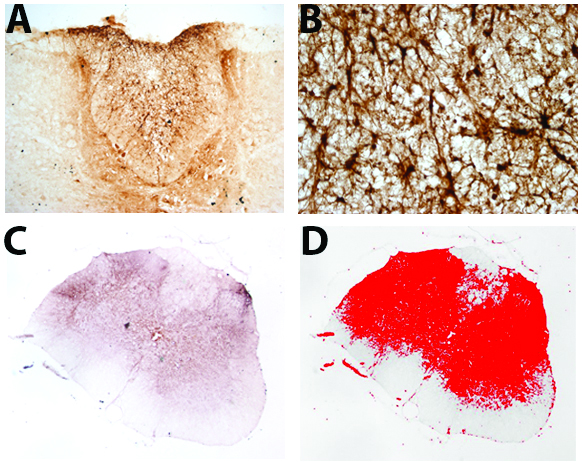

Supplement: Supplementary file 3 — Supplementary Figure 2 [file 41434_2019_75_MOESM3_ESM.jpg]

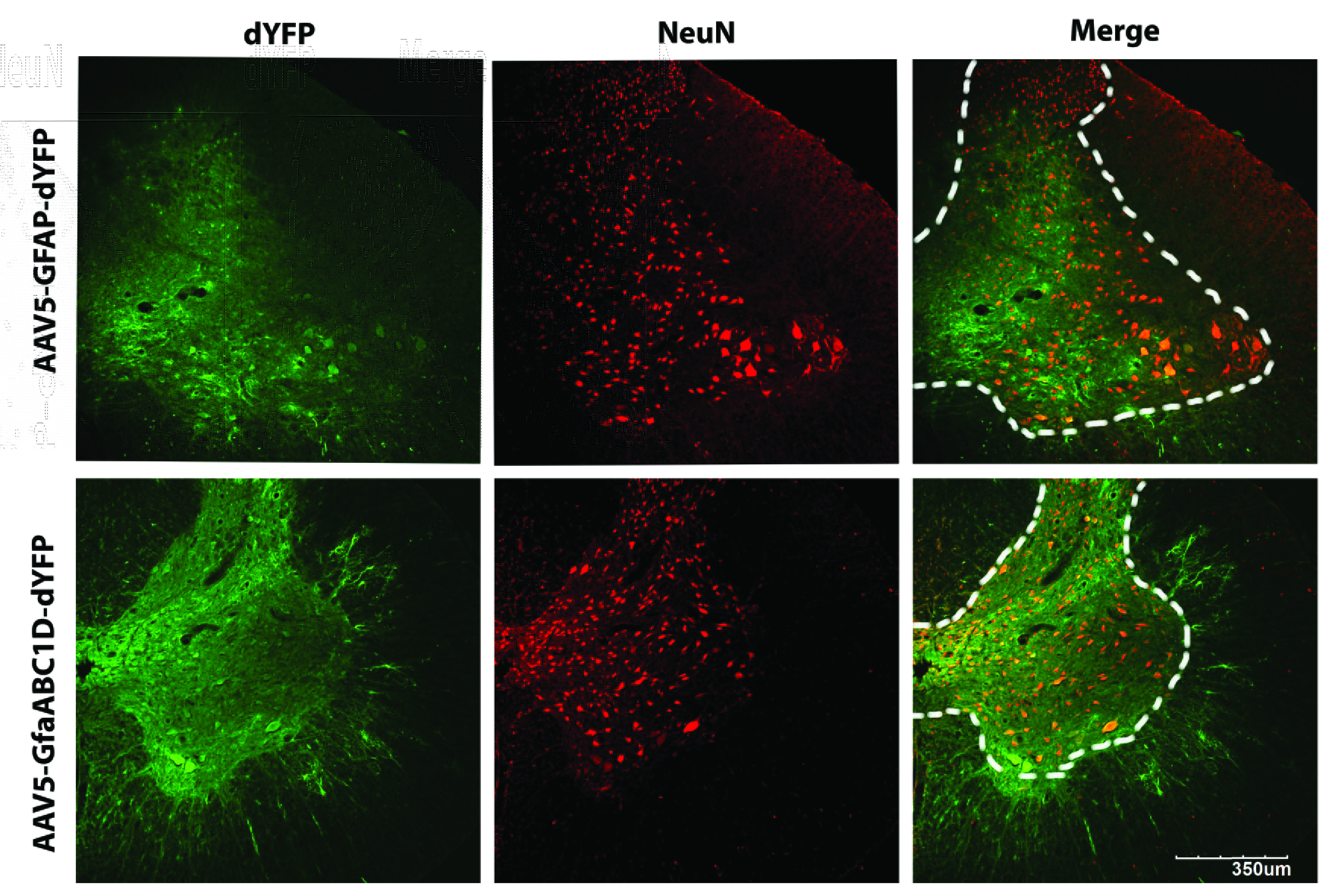

Supplement: Supplementary file 4 — Supplementary Figure 3 [file 41434_2019_75_MOESM4_ESM.jpg]
